# Paracetamol plus ibuprofen for the treatment of fever in children (PITCH): economic evaluation of a randomised controlled trial

**DOI:** 10.1136/bmj.a1490

**Published:** 2008-09-09

**Authors:** Sandra Hollinghurst, Niamh Redmond, Céire Costelloe, Alan Montgomery, Margaret Fletcher, Tim J Peters, Alastair D Hay

**Affiliations:** 1 Academic Unit of Primary Health Care, NIHR National School for Primary Care Research, Department of Community Based Medicine, University of Bristol, Bristol BS8 2AA; 2 Faculty of Health and Social Care, University of the West of England, Bristol BS16 1QY

## Abstract

**Objective** To estimate the cost to the NHS and to parents and carers of treating febrile preschool children with paracetamol, ibuprofen, or both, and to compare these costs with the benefits of each treatment regimen.

**Design** Cost consequences analysis and cost effectiveness analysis conducted as part of a three arm, randomised controlled trial.

**Participants** Children between the ages of 6 months and 6 years recruited from primary care and the community with axillary temperatures ≥37.8°C and ≤41°C.

**Interventions** Paracetamol, ibuprofen, or both drugs.

**Main outcome measures** Costs to the NHS and to parents and carers. Cost consequences analysis at 48 hours and 5 days comparing cost with children’s temperature, discomfort, activity, appetite, and sleep; cost effectiveness analysis at 48 hours comparing cost with percentage of children “recovered.”

**Results** Difficulties in recruiting children to the trial lowered the precision of the estimates of cost and some outcomes. At 48 hours, cost to the NHS was £11.33 for paracetamol, £8.49 for ibuprofen, and £8.16 for both drugs. By day 5 these costs rose to £19.63, £18.36, and £13.92 respectively. For parents and carers, the 48 hour costs were £23.86 for paracetamol, £20.60 for ibuprofen, and £25.07 for both, and the day 5 costs were £26.35, £29.90, and £24.02 respectively. Outcomes measured at 48 hours and 5 days were inconclusive because of lack of power; the cost effectiveness analysis at 48 hours provided little evidence that one treatment choice was significantly more cost effective than another. At 4 hours ibuprofen and the combined treatment were superior to paracetamol in terms of the trial primary outcome of time without fever; at 24 hours the combined treatment performed best on this outcome.

**Conclusions** There is no strong evidence of a difference in cost between the treatments, but clinical and cost data together indicate that using both drugs together may be most cost effective over the course of the illness. This treatment option performs best and is no more expensive because of less use of healthcare resources, resulting in lower costs to the NHS and to parents.

## Introduction

Fever is a common symptom of many childhood illnesses. Although these are often self limiting, the fever itself can cause distress and discomfort to the child and anxiety to parents. Over the counter preparations are available to relieve fever and its associated symptoms; they are used extensively, but the best treatment remains unclear. European expenditure on over the counter purchases of paracetamol (acetaminophen) for children has fallen between 1997 and 2004 (from £188m to £128m), whereas the use of ibuprofen has increased during the same period (from £65m to £277m, personal communication Boots Health Care). Many parents consult the National Health Service (NHS) for advice through general practices, walk-in centres, and NHS Direct. One consequence of this is widespread prescribing of antipyretics for children; for example, in 2004 prescriptions for oral suspensions of paracetamol and ibuprofen cost about £3.5m in England alone.^1^ Paracetamol and ibuprofen are increasingly used together for the relief of fever and its associated symptoms, though it is not known whether a combination of both is superior to either single drug. One study that compared these treatments collected some data on resource use, but these were not valued.^2^ To our knowledge, the cost implications have not been previously investigated.

The aim of this study was to estimate the cost to the NHS and to parents and carers of treating preschool children with a fever with paracetamol, ibuprofen, or both drugs. These costs were compared with the benefits of each treatment regimen.

## Methods

This evaluation forms part of a randomised controlled trial conducted in Bristol, as described in our associated paper.^3^ Children between the ages of six months and six years with a temperature of at least 37.8°C and no higher than 41°C were recruited to the study between January 2005 and May 2007 by one of three methods. Local and remote recruitment was from general practices, out of hours cooperatives, NHS Direct, a walk-in centre, and the emergency department of a children’s hospital. Children were also recruited direct from the community. Participants were randomised to one of three treatments options: paracetamol alone, ibuprofen alone, or a combination of both. Placebo drugs were used to blind parents and researchers to the allocated treatment. These drugs were administered regularly for the first 24 hours, at the maximum dose appropriate for the child’s weight.^3^ Between 24 and 48 hours parents gave the drugs as required, depending on the child’s symptoms.

The primary outcomes of “time without fever” and discomfort were measured in the first four hours and at 48 hours respectively. Additional secondary outcomes including temperature, activity, appetite, and sleep were measured at different time points across the five day follow-up period.

### Study design

The economic evaluation was conducted from the perspectives of the NHS and of parents and carers. We included all relevant resource use during the five days after randomisation, excluding the consultation at which recruitment to the study took place, if applicable. Costs to the NHS included practice based consultations with a doctor or nurse, telephone consultations, visits to a walk-in centre, contacts with NHS Direct, out of hours care, visits to accident and emergency departments, inpatient hospital care, ambulance use, and prescribed medication.

For the parents and carers, direct costs included travel to healthcare facilities for visits associated with the child’s fever, over the counter drugs purchased, extra care for dependents required because of the child’s illness, and loss of earnings as a result of the child’s illness. We also included a cost representing the over the counter cost of the study drugs as most antipyretics in the United Kingdom are bought rather than prescribed.

### Cost consequences analysis

We carried out a cost consequences analysis at 48 hours and five days from baseline for the NHS and for parents and carers. We recorded a range of benefits at both time points, including the child’s temperature, discomfort, activity, appetite, and sleep. These were treated as individual outcomes and also combined to provide an indication of whether the child had “fully recovered.” This was based on parents reporting that the child was “normal” for him or her with respect to discomfort, activity, appetite, and sleep and on the child having a temperature <37.2°C. Thus “fully recovered” is in effect “returned to normal for that child.”

### Cost effectiveness analysis

We used the combined outcome of “fully recovered” in a cost effectiveness analysis at 48 hours to estimate the cost per extra child returning to “normal for that child” and to indicate the relative efficiency of each treatment at a point before it was anticipated that most children would have recovered fully.

### Data collection and unit costs

A research nurse collected data from parents and carers on resource use and out of pocket expenses by means of a face to face questionnaire at 48 hours and by telephone at day 5. Table 1 shows the source of costing and unit costs. We valued primary care contacts in accordance with Curtis and Netten,^4^ and we used the NHS tariff^5^ and Department of Health reference costs^6^ for secondary care and ambulance services. We valued visits to the walk-in centre and contact with NHS Direct in accordance with information from published national evaluations.^7^ ^8^ For prescribed drugs, we used the costs reported in the *British National Formulary*,^9^ and we used the AA schedule of motoring costs^10^ for valuing travel by car. Parents who reported loss of income were asked how many days of work had been affected, and this was valued using a national wage rate.^11^ We costed the study drugs as though parents had bought them over the counter. Throughout the trial children received doses of study medicines according to their weight. We costed the consumption of medicines accordingly. All resources were valued in pounds sterling at 2006 prices using an appropriate inflation index.^4^

**Table 1 tbl1:** Resources (and their unit costs) considered in economic analysis of treating febrile preschool children with paracetamol, ibuprofen, or both

Resource	Unit cost (£)
Primary care^4^:	
General practitioner at surgery	21.00
General practitioner by telephone	23.00
Practice nurse	8.00
Health visitor	24.83
Out of hours^15^:	
Nurse telephone	12.00
Doctor telephone*	34.50
Doctor face to face†	31.50
Walk-in centre^7^	29.81
NHS Direct^8^	18.55
Accident and emergency department^5^	71.00
Inpatient stays^5^:	
Pneumonia	1063.00
Bronchiolitis	942.00
Upper respiratory tract infection	550.00
Ambulance^6^	132.90
Study drugs‡:	
Paracetamol (100 ml)	2.48
Ibuprofen (100 ml)	4.13
Study drugs for sensitivity analysis§:	
Paracetamol (100 ml)	0.42
Ibuprofen (100 ml)	2.69
Travel costs (per mile)^10^	0.49
Lost income per day^11^	94.80

^*^Based on a 10 minute consultation.

†Based on a 10.8 minute consultation.

‡Mean cost reported by parents buying these over the counter between 48 hours and five days after baseline.

§Cost if prescribed (from *British National Formulary*^9^).

### Data analysis

We estimated frequencies of resource use by the patients in each treatment arm, and mean cost per patient in each arm. We excluded inpatient care and use of ambulances in the base case analysis as these are unusual in a primary care population and could be unequally distributed by chance. Bootstrapping (1000 replicates) was used to estimate cost effectiveness planes and cost effectiveness acceptability curves to indicate the level of uncertainty around the point estimates of the incremental cost effectiveness ratios.

It was not necessary to discount the costs and outcomes, as the time horizon of the study was five days. All analyses were carried out with Microsoft Excel and Stata 9.

### Sensitivity analysis

We tested the robustness of our results against three possible areas of subjectivity. Firstly, we re-estimated the cost per patient from both perspectives if the study drugs had been prescribed rather than purchased over the counter. Secondly, we investigated the effect on the results if dosing had been by age rather than weight. Thirdly, we estimated the cost of hospitalisations.

## Results

A total of 156 children were recruited to the study—45 (29%) through local recruitment, 84 (54%) by the remote method, and 27 (17%) directly from the community. Of the 52 children in each of the three treatment groups, those in the combined treatment arm were slightly younger and weighed less than the others (mean age 25.1 months *v* 28.7 months for the paracetamol only group and 28.1 months for the ibuprofen only group; mean weight 12.6 kg *v* 13.0 kg and 13.4 kg respectively). There were more boys than girls (87 *v* 69), and this imbalance was most concentrated in the ibuprofen only group (37 (71%) boys). Most (64%) had a non-specific viral illness or a respiratory tract infection, 16% had otitis media, and the remaining 20% were assigned a variety of other diagnoses.

Data on NHS costs were complete for 154 (99%) children at 48 hours and 150 (96%) at five days. Personal costs were reported by 143 (92%) parents at 48 hours and 130 (83%) at day 5.

### Resource use

Table 2 shows the mean resource use per child over the five day follow-up. Sixty per cent of children (93) used no extra NHS resources after the consultation at which they were recruited to the study and 71% (109) had no contact with their general practitioner. Most (52%) primary care contacts were face to face at the surgery, 14 (16%) were with the out of hours service, and the rest were either telephone consultations or with a variety of other primary care providers such as walk-in centres or NHS Direct. Children receiving paracetamol had fewest face to face consultations, though most consultations overall, but there was no significant difference in total use of primary care resources among the three groups. 

**Table 2 tbl2:** Resource use associated with treating febrile preschool children with paracetamol, ibuprofen, or both. Values are mean (SD) use per episode per child for five day follow-up

Item of resource use	Paracetamol			Ibuprofen			Paracetamol + ibuprofen	
	n	Mean (SD)		n	Mean (SD)		n	Mean (SD)
Primary care consultations at surgery	51	0.24 (0.51)		50	0.30 (0.51)		52	0.42 (0.61)
Primary care telephone consultations	52	0.08 (0.27)		50	0.06 (0.24)		52	0.04 (0.19)
Out of hours consultations	52	0.15 (0.50)		50	0.10 (0.10)		52	0.06 (0.31)
Other primary care consultations	52	0.19 (0.19)		50	—		52	0.04 (0.20)
Prescribed drugs (No of items)	52	0.27 (0.56)		50	0.24 (0.52)		52	0.19 (0.44)
Over the counter drugs (No of items):								
Excluding study drugs	52	0.44 (0.73)		50	0.36 (0.63)		52	0.52 (0.85)
Including study drugs	52	1.19 (0.40)		50	1.16 (0.37)		52	2.08 (0.27)
A&E visits	51	0.04 (0.20)		50	0.10 (0.42)		52	0.04 (0.19)
Inpatient hospital stays (No of nights)	51	0.02 (0.14)		49	0.12 (0.60)		52	0.08 (0.56)
Ambulance use (No of journeys)	51	—		50	0.06 (0.31)		52	0.02 (0.14)
Days off work (No of days)	52	0.60 (1.12)		49	0.77 (1.44)		52	0.44 (0.80)
Loss of income (proportion of parents incurring a cost)	52	0.10 (0.30)		52	0.19 (0.40)		52	0.12 (0.32)
Childcare cost (proportion of parents incurring a cost)	52	0.04 (0.19)		52	0.08 (0.27)		52	0.06 (0.24)

n=Number of patients included in analysis. A&E=Accident and emergency department.

Thirty six prescriptions were issued (excluding two for drugs that had been provided in the study). Most (81%) were for antibiotics. Of the 113 over the counter preparations that were purchased (for 46 children), 62 (55%) were for paracetamol or ibuprofen, and 29 (47%) of these were bought in the first 48 hours (when study drugs were provided) and 24 (83%) were for the active ingredient being provided. 

Five children spent some time in hospital. Ninety two days of work were lost among 48 (31%) parents because of their child’s illness, and 21 (44%) of these reported a direct loss of earnings. Nine (6%) parents incurred out of pocket expenses for sibling or other dependent care because of the child’s illness.

### Cost analysis

Table 3 shows the mean cost per patient, by treatment group, during the first 48 hours and over the five day follow-up. About 60% of all NHS costs are accounted for by general practitioner appointments, and visits to accident and emergency departments was the second largest contributor. Personal costs were dominated by loss of income.

**Table 3 tbl3:** Costs (£) associated with treating febrile preschool children with paracetamol, ibuprofen, or both. Values are mean (SD) cost per child by treatment group

Cost item	0-48 hours (intervention period)				0-5 days (total follow-up)		
	Paracetamol	Ibuprofen	Paracetamol + ibuprofen		Paracetamol	Ibuprofen	Paracetamol + ibuprofen
**NHS costs**	**(n=51)**	**(n=52)**	**(n=51)**	****	**(n=50)**	**(n=49)**	**(n=51)**
Primary care doctor consultations	6.15 (15.41)	3.99 (10.67)	6.48 (13.36)		12.10 (28.30)	10.38 (18.17)	10.23 (14.67)
Primary care nurse consultations	0	0.15 (1.11)	0		0.58 (4.09)	0.16 (1.14)	0
Other primary care consultations	2.03 (7.29)	0	0		3.55 (9.37)	0	0.36 (2.60)
Total primary care cost	8.18 (17.26)	4.14 (11.16)	6.48 (13.36)		16.23 (34.11)	10.54 (18.42)	10.59 (15.16)
A&E	2.78 (13.92)	4.10 (21.84)	1.39 (9.94)		2.84 (14.05)	7.24 (29.86)	2.78 (13.92)
Prescribed drugs	0.37 (1.00)	0.25 (0.85)	0.29 (0.86)		0.56 (1.27)	0.58 (1.43)	0.55 (1.63)
Total NHS cost	11.33 (23.18)	8.49 (29.13)	8.16 (16.36)		19.63 (38.11)	18.36 (40.26)	13.92 (23.17)
**Parental costs**	**(n=47)**	**(n=49)**	**(n=47)**	****	**(n=45)**	**(n=42)**	**(n=43)**
Travel cost	0.31 (1.04)	0.02 (0.08)	0.21 (0.74)		0.70 (1.56)	0.29 (0.77)	0.35 (0.89)
Over the counter drugs*	2.52 (0.29)	4.13 (0.00)	6.75 (0.68)		3.69 (1.61)	4.74 (1.44)	7.96 (2.29)
Other expenditure	21.03 (62.18)	16.44 (58.50)	18.10 (51.64)		21.97 (63.41)	24.83 (90.81)	15.64 (46.74)
Total parental costs	23.86 (62.20)	20.60 (58.52)	25.07 (51.60)		26.35 (63.37)	29.90 (90.68)	24.02 (46.36)

A&E=Accident and emergency department.

*Includes cost of study drugs as if parents had bought over the counter.

### Cost consequences analysis: 48 hours and 5 days

Table 4 shows the cost consequences matrix. From the perspective of the NHS, the combined drug treatment was cheapest at both 48 hours and 5 days. Paracetamol only was the most expensive. Ibuprofen only was cheapest to parents at 48 hours, but by day 5 the combined treatment had become less expensive because the greater parental spending on drugs was offset by lower travel costs (because of less health service use) and less time off work.

**Table 4 tbl4:** Cost consequences analysis associated with treating 156 febrile preschool children with paracetamol, ibuprofen, or both

Costs and outcomes	0-48 hours (intervention period)				0-5 days (total follow-up)		
	Paracetamol + ibuprofen *v* paracetamol	Paracetamol + ibuprofen *v* ibuprofen	Ibuprofen *v* paracetamol		Paracetamol + ibuprofen *v* paracetamol	Paracetamol + ibuprofen *v* ibuprofen	Ibuprofen *v* paracetamol
**Incremental mean (95% CI) cost per patient (£)**							
NHS costs	−3.16 (−11.0 to 4.7)	−0.33 (−9.6 to 8.9)	−2.84 (−13.1 to 7.5)		−5.71 (−18.1 to 6.7)	−4.44 (−17.4 to 8.5)	−1.27 (−16.9 to 14.4)
Parental costs	1.20 (−22.2 to 24.6)	4.47 (−17.9 to 26.9)	−3.27 (−27.7 to 21.2)		−2.33 (−26.0 to 21.3)	−5.88 (−36.8 to 25.1)	3.55 (−29.6 to 36.7)
Outcomes^*^							
Temperature (°C): adjusted difference (95% CI) between means	0.21 (−0.20 to 0.61)	0.23 (−0.18 to 0.64)	−0.02 (−0.46 to 0.41)		−0.14 (−0.51 to 0.22)	−0.08 (−0.45 to 0.28)	−0.06 (−0.45 to 0.34)
Discomfort: odds ratio (95% CI) of “well” *v* “unwell”	1.33 (0.49 to 3.56)	0.89 (0.32 to 2.43)	1.50 (0.53 to 4.26)		0.45 (0.13 to 1.59)	0.75 (0.24 to 2.34)	0.60 (0.15 to 2.39)
Activity: odds ratio (95% CI) of “well/normal” *v* “unwell/not normal”	0.67 (0.26 to 1.70)	0.40 (0.13 to 1.20)	1.68 (0.60 to 4.67)		0.30 (0.08 to 1.10)	0.45 (0.14 to 1.49)	0.66 (0.15 to 2.99)
Appetite: odds ratio (95% CI) of “well/normal” *v* “unwell/not normal”	1.08 (0.41 to 2.84)	0.80 (0.30 to 2.01)	1.39 (0.50 to 3.82)		1.16 (0.45 to 2.94)	1.07 (0.42 to 2.73)	1.10 (0.40 to 2.91)
Sleep: odds ratio (95% CI) of “well/normal” *v* “unwell/not normal”	0.84 (0.34 to 2.07)	0.56 (0.22 to 1.40)	1.49 (0.57 to 3.92)		0.64 (0.25 to 1.62)	1.09 (0.44 to 2.71)	0.59 (0.17 to 1.35)
Difference (95% CI) in proportion of children returning to “normal for that child”	−0.06 (−0.23 to 0.11)	−0.04 (−0.22 to 0.13)	−0.02 (−0.20 to 0.16)		−0.04 (−0.23 to 0.15)	0.04 (−0.15 to 0.23)	−0.08 (−0.27 to 0.11)

^*^Adjusted for minimisation variables: age, temperature, fever duration, antibiotic use and discomfort.

The results for the children’s temperature, discomfort, activity, appetite, and sleep suggest that ibuprofen alone may perform best at 48 hours, but the wide confidence intervals around all incremental costs and outcomes indicate only weak evidence in support of any differences.

### Cost effectiveness analysis at 48 hours

Table 5 presents the incremental costs and benefits at 48 hours. Cost is expressed as incremental mean cost per child by treatment group; benefits are expressed as the proportion of children per group returning to normal. From the NHS perspective, the combined treatment is cheaper but (slightly) less effective than either of the two monotherapies, paracetamol alone is more expensive and marginally more effective than ibuprofen alone. 

**Table 5 tbl5:** Cost effectiveness at 48 hours for treating febrile preschool children with paracetamol, ibuprofen, or both

	Paracetamol	Ibuprofen	Paracetamol + ibuprofen	Paracetamol + ibuprofen *v* paracetamol	Paracetamol + ibuprofen *v* ibuprofen	Ibuprofen *v* paracetamol
**NHS perspective (n=154)**						
Mean (SD) total cost (£)	11.33 (23.18)	8.49 (29.13)	8.16 (16.36)	—	—	—
Incremental cost (95% CI) (£)	—	—	—	−3.16 (−11.05 to 4.72)	−0.33 (−9.59 to 8.93)	−2.84 (−13.14 to 7.46)
Proportion (SD) of children recovering*†	0.27 (0.45)	0.27 (0.45)	0.24 (0.43)	—	—	—
Incremental benefit (95% CI)	—	—	—	−0.04 (−0.21 to 0.13)	−0.03 (−0.21 to 0.14)	−0.01 (−0.18 to 0.17)
Cost per extra child recovering†	—	—	—	£80.70	£9.62	£537.65
**Parent perspective (n=143)**						
Mean (SD) total cost (£)	23.86 (62.20)	20.60 (58.52)	25.07 (51.60)	—	—	—
Incremental cost (95% CI) (£)	—	—	—	1.20 (−22.20 to 24.60)	4.47 (−17.90 to 26.90)	−3.27 (−27.70 to 21.20)
Proportion (SD) of children recovering†	0.30 (0.46)	0.29 (0.46)	0.23 (0.43)	—	—	—
Incremental benefit (95% CI)	—	—	—	−0.06 (−0.25 to 0.12)	−0.05 (−0.23 to 0.13)	−0.01 (−0.20 to 0.17)
Cost per extra child recovering†	—	—	—	£−18.87	£−86.55	£268.78

*Based on complete cases: proportions vary by perspective due to missing data.

†Recovering is defined as “returning to normal for that child.”

From the parental perspective, paracetamol and ibuprofen together is more expensive and less effective than either of the single treatments. Ibuprofen alone is cheaper but less effective than paracetamol alone. The level of uncertainty around the incremental cost effectiveness ratios is shown by the bootstrapped replications shown on the cost effectiveness planes in fig 1. Replications fall in all four quadrants for all comparisons, suggesting there is little evidence that any treatment choice is significantly more cost effective than any other. This is reinforced by the cost effectiveness acceptability curves in fig 2, where none of the probabilities of one treatment being more cost effective than another reaches 50%.

**Figure fig1:**
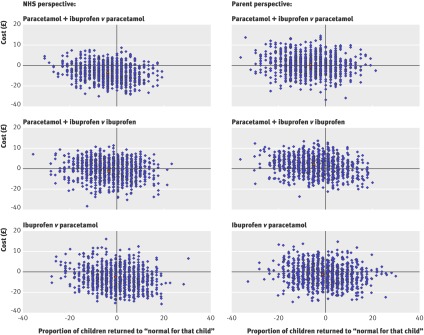
**Fig 1** Cost effectiveness planes comparing cost of treating fever in preschool children with the proportion of children who have returned to “normal for that child” at 48 hours

**Figure fig2:**
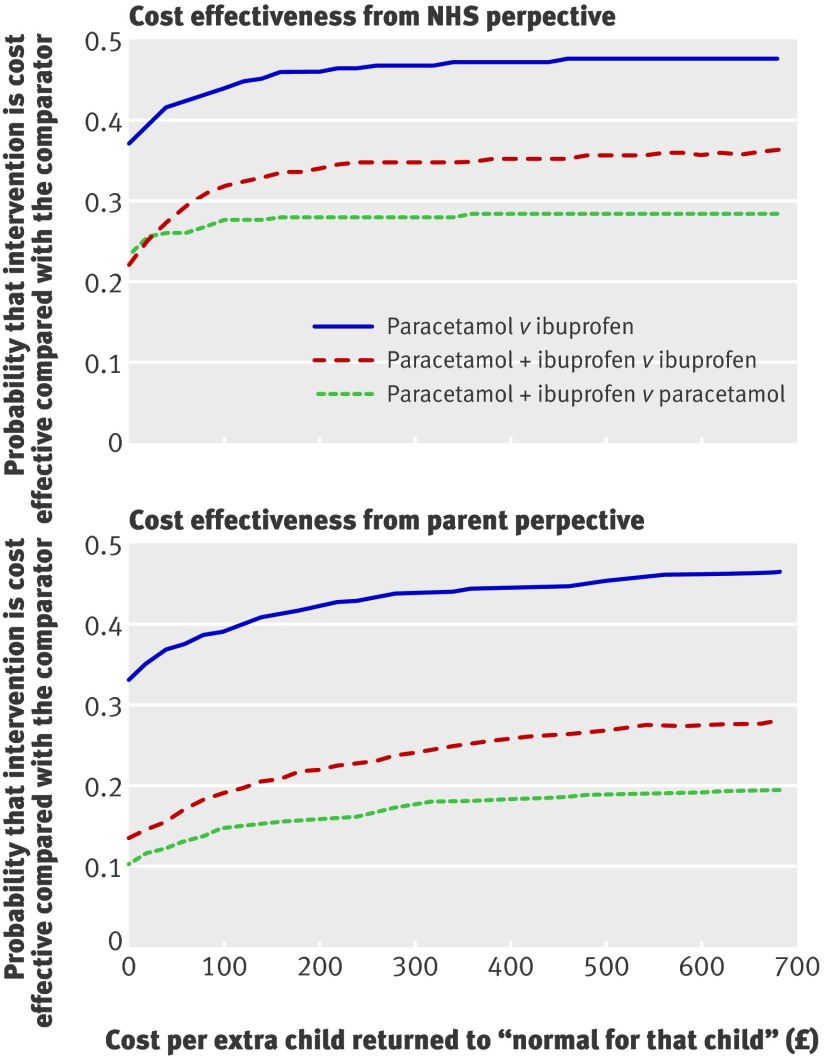
**Fig 2** Cost effectiveness acceptability curves from NHS and parent perspectives of treating fever in preschool children at 48 hours

### Sensitivity analysis

Table 6 gives the results of the three sensitivity analyses for different scenarios. For each one-way analysis, we show the mean (SD) cost for the NHS and for parents based on the revised assumptions. We also give the new incremental cost and the change in the incremental cost from the base case results. Firstly, if the study drugs were prescribed rather than bought over the counter, costs to the NHS increase but by less than the associated decrease in parental costs. The increase in NHS costs is greatest for the combined treatment, though this is still the cheapest treatment option for the NHS at day 5.

**Table 6 tbl6:** Sensitivity analyses for different scenarios on costs associated with treating febrile preschool children with paracetamol, ibuprofen, or both

Different scenarios	Mean (SD) costs				Incremental cost (change from base case)		
	Paracetamol	Ibuprofen	Paracetamol + ibuprofen		Paracetamol + ibuprofen *v* paracetamol	Paracetamol + ibuprofen *v* ibuprofen	Ibuprofen *v* paracetamol
**If study medicines had been prescribed**							
For 0-48 hours:							
NHS costs	11.69 (22.98)	11.18 (29.13)	11.27 (16.36)		−0.42 (2.74)	0.09 (0.42)	−0.52 (2.32)
Parental costs	21.88 (62.09)	16.87 (59.89)	19.30 (51.39)		−2.58 (−3.78)	2.43 (−2.04)	−5.01 (−1.74)
For 0-5 days:							
NHS costs	20.00 (37.99)	20.92 (39.87)	17.02 (23.18)		−2.98 (2.73)	−3.91 (0.53)	0.92 (2.20)
Parental costs	22.88 (63.27)	25.28 (90.81)	16.14 (46.68)		−6.73 (−4.13)	−9.14 (−2.48)	2.40 (−1.65)
**If dose by age**							
Parental costs for 0-5 days	25.36 (63.27)	29.41 (90.81)	22.75 (46.68)		−2.60 (−0.28)	−6.66 (−0.78)	4.05 (0.50)
**Including secondary care costs**							
NHS costs for 0-5 days	40.89 (164.8)	78.64 (268.3)	35.19 (168.6)		−5.70 (0.01)	−43.45 (−39.01)	37.75 (39.02)
Parental costs for 0-5 days	26.89 (63.23)	31.21 (90.6)	24.28 (46.3)		−2.60 (−0.27)	−6.92 (−0.26)	4.32 (0.30)

Secondly, if parents dosed their child by age (as per the instructions on the bottle) rather than by weight (as in this study) then the parents of 45 children (29%) would have used fewer bottles of medicine. Although about half (51%) of these children were in the paracetamol only group, the cost impact is greatest in the combined treatment group because two drugs have to be purchased and ibuprofen is more expensive than paracetamol.

Finally, we included secondary care costs (inpatient care, ambulance use, and travel cost for families). These are estimated at day 5 only as the episodes of care generally spanned the whole period. Of the five children who received secondary care, one was in the paracetamol only group, one in the combined treatment group, and three were in the ibuprofen only group. This is reflected in the cost estimates, which increase by about £20 per hospitalised child per group (that is, about £1000 per hospitalisation). There is no evidence that any of these events was related to the study drugs the child received, and the combined treatment remains the most attractive choice for the NHS and for parents.

## Discussion

Over the course of five days the mean cost of care for a preschool child with fever was estimated to be £27 to parents and carers and £17 to the NHS, excluding the cost of any index consultation. Two thirds of the NHS costs were due to consultations with general practitioners. Taken together, the results of our present study and those reported in our trial paper^2^ suggest that paracetamol and ibuprofen given in combination is more effective at 24 hours than either drug given alone and possibly cheaper over a five day period.

### Strengths and weaknesses of the study

To our knowledge, this is the first investigation of the cost effectiveness of dual antipyretic drugs versus single drug use. This economic evaluation benefited from being part of a randomised controlled trial. Data collection and entry were thorough and rigorously checked; and data quality was enhanced by our method of collection: the research nurses spoke to parents face to face or by telephone. For the same reason, the level of missing data was minimal.

We are aware of several limitations. Because of recruitment problems, we were unable to achieve our original target sample size.^2^ This affected interpretation of the cost data, and some of the outcome data as the study was eventually powered to detect clinical differences solely in the time spent without fever. This outcome was measured at 4 hours and 24 hours, but cost data were not collected for this short time period. We were underpowered with respect to the outcomes measured or reported at 48 hours and five days, when cost data were collected.

The cost effectiveness analysis at 48 hours was based on a combined measure of child’s temperature, discomfort, activity, appetite, and sleep—from which we defined “recovered” to mean “normal for that child.” As these outcomes were affected by the lack of power, our choice of outcome for the cost effectiveness analysis limits its value. None of the comparisons showed evidence of differences between the treatments in terms of cost effectiveness, and it is therefore difficult to draw strong conclusions from this part of the analysis.

The economic evaluation was intended to enhance the associated clinical study^3^ by taking a longer term view and provide information about costs and benefits over the whole episode of illness. We chose a five day follow-up period because we expected that most children would have recovered by this time. In fact, this was not the case; according to our strict definition, only 36% children had recovered by five days, mainly because their appetite and sleep had not returned to normal. Eating and sleeping patterns seem to remain disrupted after an illness for longer than we originally hypothesised, but it is unlikely that these effects would result in significant further use of healthcare resources as nearly 90% children had a normal temperature and no discomfort at this point. A further, related, limitation is lack of evidence about any long term adverse effects and allied costs of using either or both antipyretic drugs for childhood fever.

These limitations show the value of presenting a cost consequences matrix of disaggregated results, which may be more valuable when different perspectives and different outcomes are of interest to different stakeholders. Although the results presented here can only be indicative because of the lack of power, they endorse our clinical findings.^3^

Finally, although we were able to estimate the direct cost to parents of time off work, we were not able to estimate the monetary value to society of that lost productivity. Such valuations are contentious and particularly so in a case such as this when the individual time off work is small but the total at a population level could be considerable. The true cost of this childhood illness is therefore likely to be greater than estimated here.

### Comparison with other literature

Our results are similar to those of Sarrell et al, who reported lower resource use in children receiving paracetamol and ibuprofen together compared with those receiving either drug on its own.^2^ We have found no published economic evaluations comparing single and dual treatment for childhood fever, though we can assess the face validity of the results of our economic analysis by looking at the cost of illness. In this study, the mean cost of an episode of illness over five days was £38 to the NHS (allowing for the cost of the initial consultation), and £27 to parents and carers. A recent cost of illness analysis estimated the cost of an episode of childhood cough to be £25 to the NHS and £15 to parents.^12^ Fever resulted in greater use of healthcare resources across the board, the difference being most marked in the use of accident and emergency departments, out of hours care, the purchase of over the counter drugs, and the effect on parental time off work.

### Meaning of the study

This economic evaluation adds to the findings of our clinical study by providing information on the relative cost of the alternative treatments over a longer time period. The results show that, over the course of the whole illness, treating children with both drugs may lead to less use of other healthcare resources than does either of the drugs alone. This would result in lower costs to the NHS and to parents because of less travel and time off work. 

Both paracetamol and ibuprofen are relatively cheap drugs, but fever is common among young children, and the population effect of using the most appropriate treatment could be substantial. About 70% of preschool children have a fever each year,^13^ and professional help is sought for about 60% of them, resulting in over 1.5 million consultations.^14^ Hence even a small reduction in cost per case could have a large impact on overall budgets. The results of this study provide some guidance on the preferred treatment for preschool children with a fever. This could produce substantial savings in the current cost burden, which mainly falls on primary care and parents. Furthermore, reducing uncertainty about the optimal treatment lessens the need for parents to consult their general practitioner for advice, thus generating further savings.

**What is already known on this topic**

Fever is a common symptom of many childhood illnesses
Paracetamol and ibuprofen are often used, separately or together, to reduce temperature and relieve symptoms, but the optimal treatment regimen in terms of cost and outcomes is unclear

**What this study adds**

Cost of care of children with a fever is largely borne by parents and the primary care sector
Over the course of five days, using paracetamol and ibuprofen together may lead to less use of healthcare resources than either drug alone, making the combined treatment the best value option

## Notes

We thank the Avon, Gloucestershire and Wiltshire NHS Direct, the Bristol general practices, the South Bristol Walk-In Centre, the Bristol Royal Hospital for Children’s Emergency Department, and the children and parents whose participation made this study possible. We thank the South West Medicines for Children Local Research Network (MCRN); the research nurse team W Horseman, J Farrimond, R Powell, S Shatford, P Richards, and MCRN nurse V Payne; W Patterson (trial coordinator); S Doohan and S Burke (project administrators); K Schroeder, M Weiss, and A Emond (co-applicants); and K Pitcher (data entry/quality). We thank the Trial Steering Committee (AL Kinmonth, C Butler, J Peacock, M Blythe, and P Denyer) and Data Monitoring and Safety Committee (R Bragonier, S Kerry, and J Chudleigh) for their support.

Ethical approval: The study was approved by the Bath Research Ethics Committee, UK (reference number 04/Q2001/197).

## Notes

**Cite this as:** *BMJ* 2008;337:a1490
